# Emulgels Containing *Perilla frutescens* Seed Oil, *Moringa oleifera* Seed Oil, and Mixed Seed Oil: Microemulsion and Safety Assessment

**DOI:** 10.3390/polym14122348

**Published:** 2022-06-09

**Authors:** Prakairat Tunit, Chuda Chittasupho, Kusuma Sriyakul, Parunkul Tungsuruthai, Panlop Chakkavittumrong, Kesara Na-Bangchang, Somboon Kietinun

**Affiliations:** 1 Graduate Program in Integrative Medicine, Chulabhorn International College of Medicine, Thammasat University, Pathum Thani 12120, Thailand; prakairat.tun@mail.pbru.ac.th (P.T.); kusumas@tu.ac.th (K.S.); parunkul@hotmail.com (P.T.); 2 Department of Pharmaceutical Sciences, Faculty of Pharmacy, Chiang Mai University, Chiang Mai 50200, Thailand; 3 Division of Dermatology, Department of Medicine, Faculty of Medicine, Thammasat University, Pathum Thani 12120, Thailand; panlop078@gmail.com; 4 Center of Excellence in Molecular Biology and Pharmacology of Malaria and Cholangiocarcinoma, Graduate Studies, Chulabhorn International College of Medicine, Thammasat University, Pathum Thani 12120, Thailand; kesaratmu@yahoo.com; 5 Graduate Program in Bioclinical Sciences, Chulabhorn International College of Medicine, Thammasat University, Pathum Thani 12120, Thailand

**Keywords:** *P. frutescens*, *M. oleifera*, mixed seed oil, microemulsion, emulgel

## Abstract

*P. frutescens* seed oil and *M. oleifera* seed oil consist of fatty acids and sterols that are beneficial for skin. Mixing of these oils at 1:1 ratio has shown to increase antioxidant activity of oils. This study aims to formulate emulgels containing microemulsions of *P. frutescens* seed oil, *M. oleifera* seed oil, and mixed *P. frutescens* and *M. oleifera* seed oils. The chemical constituents of *P. frutescens* seed oil, *M. oleifera* seed oil, and mixed seed oil are analyzed by gas chromatography/mass spectrometry (GC/MS). The microemulsions are formulated by a phase titration method and characterized for the droplet size, polydispersity index, and zeta potential value using a dynamic light scattering technique. The physical and chemical stability of the microemulsions are investigated using a rheometer and UV-Visible spectrophotometer, respectively. The safety of microemulsion is evaluated on PBMC and human subjects. Emulgels containing three different types of microemulsion are formulated. The results show that *P. frutescens* seed oil is mainly composed of alpha-linolenic acid, linoleic acid, and oleic acid, whereas *M. oleifera* seed oil contains a high proportion of oleic acid. Mixed seed oil contains a comparable amount of alpha-linolenic acid and oleic acid. All types of oils are composed of β-sitosterol as the major plant sterol. Microemulsions of all types of oils are successfully prepared by using Tween 80 as a surfactant due to the largest transparent region of pseudoternary phase diagram. The size, polydispersity index, and zeta potential values of all types of microemulsion are in the acceptable range upon storage at 30 °C for 1 month. Microemulsions exhibit pseudoplastic flow behavior. The percent of remaining oils in all types of microemulsion is more than 90% after storage at 30 °C for 1 month. Emulgels containing three types of microemulsions exhibit good characteristics and no change in viscosity after storage at 4, 30, and 45 °C for 1 month. The safety results reveal that three types of microemulsion do not induce cytotoxicity to PBMC nor induce skin irritation and allergic reactions. Emulgels containing microemulsions developed in this study can be used to safely deliver *P. frutescens* seed oil, *M. oleifera* seed oil, and mixed seed oil to human skin.

## 1. Introduction

Cold-pressed plant oils are rich in nutrients, including fatty acids, sterols, triglycerides, tocopherols, tocotrienols, phospholipids, waxes, squalene, triterpene alcohols, hydrocarbons, polyphenols, and fat-soluble vitamins [1]. The composition of plant oils has many benefits to skin and can affect the skin barrier, inflammation, oxidation, and proliferation, depending on the type of oil and its composition [2]. Different types of saturated and unsaturated fatty acids in plant oils influence the effect on skin. *Perilla frutescens* seed oil contains a high amount of alpha-linolenic acid or omega-6 fatty acid, followed by linoleic acid or omega-3, and oleic acid. These fatty acids are shown to have many benefits to human health, including preventing cardiovascular diseases by managing cholesterol, triglyceride, and blood pressure levels, supporting mental health by preventing depression, Parkinson’s disease and psychosis, and reducing inflammation occurring in some chronic diseases. The anti-inflammatory property of alpha-linolenic acid is beneficial for blemish-prone skin [3]. Gamma-linolenic acid has antioxidant activity that can be used to protect skin from environmental damage and skin aging [4,5]. The double blinded placebo controlled clinical study investigated by Kim et al. has shown that gamma-linolenic acid can improve erythema index, melanin index, Transepidermal water loss, and stratum corneum hydration in most volunteers without serious side effects [6]. Alpha-linolenic acid and linoleic acid were shown to accelerate the turnover of the stratum corneum and played an important role in the melanin pigment removal from the epidermis. In addition, alpha-linolenic acid and linoleic acid can suppress melanin production suggesting pigment-lightening effect of these fatty acids [7]. Along with alpha-linolenic acid and linoleic acid, other polyunsaturated fatty acids were also suggested as promising therapeutic agents for the treatment of many skin disorders such as atopic dermatitis, psoriasis, acne vulgaris, systemic lupus erythematosus, non-melanoma skin cancer, and melanoma. The mechanisms underlying these pharmacological activities are maintaining stratum corneum permeability barrier, maturation and differentiation of the stratum corneum, inhibition of proinflammatory cytokines, increasing sunburn resistance, inhibition of anti-inflammatory enzymes, inducing cancer cell apoptosis, and promoting wound healing of the skin. Topical applications of alpha-linolenic acid, linoleic acid, and oleic acid can enhance the closure of wounds induced by skin surgery [8].

*Moringa oleifera* seed oil primarily contains oleic acid, which has anti-inflammatory and antioxidant, antibacterial, and antifungal activity. It can prevent cardiovascular disease by reducing cholesterol level and blood pressure and it has an antiepileptic characteristic. In addition to fatty acids, *M. oleifera* seed oil contains tocopherols, vitamin A, and polyphenols and has been used in various topical products to moisten skin [9]. Oleic acid is a major unsaturated fatty acid found in *M. oleifera* seed oil. Oleic acid is known as a permeation enhancer that interacts with the lipid matrix to disrupt lipid fluidity and reduces the diffusional resistance of the skin [10].

Blending fatty acid and other compositions can enlarge the application and produce a new product for specific biological activity. Mixing *P. frutescens* seed oil and *M. oleifera* seed oil is hypothesized to enhance effects of both oils. *P. frutescens* seed oil contains a high amount of alpha-linolenic acid and linoleic acid, which have benefits for skin repair, whereas *M. oleifera* seed oil can improve skin permeability of active nutrients in both oils [2]. Despite the benefits of the fatty acids in *P. frutescens* seed oil and *M. oleifera* seed oil, these compounds have poor bioavailability due to low absorption and low water solubility. In addition, they are susceptible to physical and chemical degradation. One of the approaches to overcome these limitations is to develop proper formulation strategies, such as encapsulation of oils in microemulsions to gain better absorption into the skin and better chemical stability [11]. Microemulsion is defined as a transparent, thermodynamic dispersion of oil and water, stabilized by an interfacial film of surfactant with/without co-surfactant. Microemulsion has advantages over conventional emulsions in terms of thermodynamical stability and spontaneous formation. Due to the good physical stability and the ability to improve the bioavailability of various drugs, microemulsion has received attention as a drug delivery system [12]. The functions of microemulsion were enhancing solubility of encapsulated compounds, protecting active compounds from physical and chemical degradation, reducing irritation, increasing absorption, improving efficacy, and decreasing toxicity of the drugs. Plant oils mostly contain non-polar compounds that have low penetration capacity, leading to reduced pharmacological activity, enhanced skin irritation, or hypersensitivity in the application area. Microemulsion offers the possibility to increase drug penetration and therapeutic efficacy and to reduce side effects.

Delivery of poorly water soluble or oil soluble drugs by passive diffusion through an intact stratum corneum is challenging [13]. Emulgel is an emulsified gel containing an aqueous phase dispersed with the lipid phase. Emulgel has shown to provide better solubility for poorly water soluble drugs and enhanced permeability. Compared with hydrogel and cream, emulgel is thixotropic, greaseless, easily spreadable, easily washable, good penetrable, and stable [14]. Emulgel can be prepared by mixing emulsion with the hydrophilic gel base. In this study, the microemulsion was added to an aqueous phase of hydrogel to formulate an emulgel. A previous study showed that emulgel loaded with flaxseed oil presented antimicrobial activities against *S. aureus*, *P. aeruginosa*, *S. pyogenes*, *E. coli*, and *K. pneumoniae*, which could be used for the treatment of diabetic foot ulcers [15].

The goal of this study is to develop and analyze the physical and chemical characteristics of the microemulsion containing *P. frutescens*, *M. oleifera*, and mixed seed oil. Emulgel containing microemulsion of *P. frutescens*, *M. oleifera*, and mixed seed oil is formulated and investigated for its physical stability. The cytotoxicity of microemulsion containing *P. frutescens* seed oil, *M. oleifera* seed oil, and mixed seed oil against the human peripheral blood mononuclear cell is determined. The safety of emulgel containing microemulsion in healthy volunteers is investigated.

## 2. Materials and Methods

### 2.1. Materials

Cold-pressed *M. oleifera* seed oil originating from India was purchased from Neo Moringa (Bangkok, Thailand). *P. frutescens* seed oil was obtained from Pang Oung local market (Meahongsorn, Thailand). Caprylic triglyceride (Lexol^®^865), polysorbate 20 (Tween 20) polysorbate 80 (Tween 80) and propylene glycol were purchased from Namsiang (Bangkok, Thailand). Roswell Park Memorial Institute 1640 medium (RPMI-1640), 1-(4,5-Dimethylthiazol-2-yl)-3,5-diphenylformazan, Thiazolyl blue formazan (MTT reagent), and histopaque-1077 were purchased from Sigma-Aldrich, St. Louis, MO, USA). Xanthan gum, glycerin, PEG/PPG-14/7 dimethyl ether, phenoxyethanol, and tocopheryl acetate were obtained from Myskinrecipe (Bangkok, Thailand).

### 2.2. Characterization of P. frutescens Seed Oil, M. oleifera Seed Oil, and Mixed Seed Oil

#### 2.2.1. Quantification of Plant Sterols in *P. frutescens*, *M. oleifera*, and Mixed Seed Oils by Gas Chromatography (GC)

Quantification of plant sterols in oil was performed using gas chromatography [16]. *P. frutescens*, *M. oleifera* seed oils, and mixed seed oil (*P. frutescens*: *M. oleifera* seed oil (1:1)) (0.5 g) were saponified with 1 mL of 50% potassium hydroxide and 4 mL of 95% ethyl alcohol with a reflux at 85 °C for 30 min. The oils were then extracted with hexane: petroleum ether (1:1). The upper layer of solution was separated and evaporated. Then, the oil extract was derivatized by dissolving with 200 µL of hexamethyldisilazane and 100 µL of trimethylchlorosilane in dimethylformamide for 15 min. Then, 1 mL of internal standard (5-α-cholestane) and distilled water was added and mixed thoroughly. The mixture was set aside until two layers were separated. The obtained upper layer solution was filtered with a nylon syringe filter. GC analysis was carried out by using a 6850 Series GC System (Agilent Technologies, Santa Clara, CA, USA) and a chemically bonded fused silica capillary column of methylsiloxane (HP-5, 30 m × 0.32 mm i.d., 0.25 µm film thickness). The injection volume was 1 µL. The inlet temperature was 280 °C, and the detector temperature was 300 °C at a flow rate of 1 mL/min with a run time of 15 min.

#### 2.2.2. Determination of the Fatty Acid Composition in *P. frutescens*, *M. oleifera*, and Mixed Seed Oils

The analysis of the fatty acid composition in all types of oils followed the compendium of methods for food analysis (National Bureau of Agriculture Commodity and Food Standards. Compendium of Methods for Food Analysis, Thailand, 2003). The oils (1 g) were extracted with 50 mL of chloroform: methanol (2:1). The supernatant was filtered. The obtained solution was evaporated by using a rotary evaporator until dry. Then, 5 mL of 0.5 M potassium hydroxide in methanol was added to the oil extract and mixed well. The internal standard (800 µg/mL tricosanoic acid methyl ester (C23:0), 1 mL) was added and placed in a water bath 100 ± 2 °C for 5 min, followed by adding 14% BF3 in methanol (2 mL) and further placing it in a water bath at 100 ± 2 °C for 15 min. The obtained solution was then extracted with petroleum ether (4 mL at a time). The upper layer of clear solution was collected and evaporated until dry and dissolved with 1 mL of n-heptane. The fatty acid composition was analyzed using a 7890 B Series GC System (Agilent Technologies, Santa Clara, CA, USA).

#### 2.2.3. Determination of Peroxide Value in *P. frutescens*, *M. oleifera*, and Mixed Seed Oils

The peroxide value of oils was determined by the iodometric titration method [17]. *P. frutescens M. oleifera* seed oils, and mixed seed oil (5 g) were dissolved in acetic acid: chloroform solution (30 mL) followed by adding potassium iodide and allowed to react for 1 min before adding 30 mL of water. Sample containing starch solution was titrated with 0.01 N sodium thiosulfate. The peroxide value was calculated using the following Equation (1).

(1) $$\mathrm{mEq}\;\mathrm{peroxide}/\mathrm{kg}\;\mathrm{fat}=\frac{\mathrm{Volume}\;\mathrm{of}\;0.01\;N\left({\mathrm{Na}}_{2}S_{2}O_{3}\right)\times 0.01\times 1000}{\mathrm{Weight}\;\mathrm{of}\;\mathrm{oil}}$$

#### 2.2.4. Thiobarbituric Acid (TBA) Test of *P. frutescens*, *M. oleifera*, and Mixed Seed Oils

The lipid peroxidation of oils was analyzed by the reaction with TBA [18]. The oils (10 g) were mixed with 97.5 mL of water and 2.5 mL of 4 M HCl. The mixture was distilled until reaching a volume of 50 mL. The obtained solution (5 mL) was mixed with 5 mL TBA and placed in a water bath at 100 °C for 35 min. The absorbance of reaction was measured at 538 nm using a UV-visible spectrophotometer (Shimadzu UV-1900i, Kyoto, Japan). TBA number was calculated as per the Equation (2).

TBA number (mg malonaldehyde/kg) = 7.8 × OD(2)

#### 2.2.5. Color Analysis

*P. frutescens* and *M. oleifera* and mixed seed oil colorimetric measurement (MSEZ-4500L, Hunter Lab, Reston, VA, USA) was carried out using a spectrophotometer. L*, a*, and b* coordinates indicated the lightness, red/green coordinate, and yellow/blue coordinate of given color, respectively.

### 2.3. Preparation of P. frutescens, M. oleifera, and Mixed Seed Oil Microemulsions

*P. frutescens*, *M. oleifera*, and mixed seed oil microemulsions were prepared by a phase titration method to obtain an optimal ratio and concentration of each component forming microemulsions First, each of the oils, namely *P. frutescens* seed oil, *M. oleifera* seed oil, and the mixed seed oil was mixed with Lexol^®^ 865 at the 1:1 ratio and the mixtures were known as Omix. Each Omix was mixed with surfactant with/without co-surfactant (Smix), including (1) Tween 20, (2) Tween 20: propylene glycol (1:1), (3) Tween 20: Span 80 (1:1), (4) Tween 80, (5) Tween 80: propylene glycol (1:1), and (6) Tween 80: Span 80 (1:1). The mixtures were then titrated with 100 µL of water for 10 times followed by adding 1000 µL of water until the total volume reached 11,000 µL. The oils, surfactant/co-surfactant, and water were mixed vigorously for 15 s by using a vortex mixer. The ratios of Smix, oil, and water giving transparent mixtures were marked as points in the phase diagram. The areas covered by these points were considered as the microemulsion regions. The transparency of the microemulsion was observed again after 1 week.

### 2.4. Physical Characterization and Physical Stability of P. frutescens, M. oleifera, and Mixed Seed Oil Microemulsions

#### 2.4.1. Size, Polydispersity Index, Zeta Potential, and pH Values

Dynamic light scattering technique was performed to measure hydrodynamic diameter, polydispersity index (PDI), and zeta potential of *P. frutescens*, *M. oleifera*, and mixed seed oil microemulsions using Zetasizer Nano ZS (Malvern Panalytical, Bristol, UK). The effective hydrodynamic diameter and PDI were recorded at 173° scattering angle under 25 °C. Physical stability of selected formulation was investigated in terms of droplet size polydispersity index, zeta potential, and pH by storing the microemulsions at 30 °C for 1 month. The heating/cooling cycle was performed to evaluate the microemulsion stability at extreme temperature changes, such as during transportation. This test was conducted by placing the microemulsions at 4 °C for 24 h and then incubated at 45 °C for 24 h in sequence. They were stored in these temperatures for 48 h, which accounted for one cycle. The test was performed for 6 consecutive cycles in total [19]. The size, PDI, zeta potential value, and pH values of microemulsions were determined every two cycles.

#### 2.4.2. Rheology Study

The rheological properties of oils and microemulsions after fresh preparation, a heating–cooling cycle stability study (6 cycles), and 1-month storage at 4 °C, 30 °C, and 45 °C were investigated using a rheometer (HAAKETM, Thermo Scientific, Dreieich, Germany). For heating–cooling cycle stability study, viscosity and rheology of samples were determined every two cycles. Flow properties were investigated by measuring the dynamic viscosity (η) as a function of time for 50 reads in addition to measurement of viscosity as a function of shear rate (ranging from 0.1 s^−1^ to 200 s^−1^).

### 2.5. Chemical Analysis of P. frutescens, M. oleifera, and Mixed Seed Oil Microemulsions

The maximum wavelengths of *P. frutescens*, *M. oleifera*, and mixed seed oils dissolved in DMSO were determined by UV-visible spectrophotometry (Shimadzu Corp., Kyoto, Japan) in the range of 200–400 nm. *P. frutescens* seed oil (0.04–1.25% *v*/*v*), *M. oleifera* seed oil (0.04–10% *v*/*v*), and mixed seed oil (0.08–10% *v*/*v*) were prepared by 2-fold dilution in DMSO to generate calibration curves. The standard curve was plotted between the absorbance at maximal wavelengths and oil concentrations. The concentrations of *P. frutescens* and *M. oleifera* seed oils loaded in each type of microemulsions were measured by using a UV-Vis spectrophotometry at the maximum wavelength and were calculated by Equation (3). The % remaining of oils after storage at 4 °C, 30 °C, and 45 °C for 0, 0.5, and 1 month were analyzed by UV-visible spectrophotometry and were calculated by using Equation (4). Concentration of oil initially added refers to the concentration of oils added during the preparation of microemulsion. The concentration of oils loaded in the microemulsion means the concentration of oils that exist in the microemulsion after preparation.

(3) $$\mathrm{Loading}\;\mathrm{efficiency}\;(\%)=\frac{\mathrm{Concentration}\;\mathrm{of}\;\mathrm{oil}\;\mathrm{in}\;\mathrm{microemulsion}}{\mathrm{Concentration}\;\mathrm{of}\;\mathrm{oil}\;\mathrm{initially}\;\mathrm{added}}\times 100$$

(4) $$\mathrm{Remaining}\;\mathrm{of}\;\mathrm{oil}\;(\%)=\frac{\mathrm{Concentration}\;\mathrm{of}\;\mathrm{oil}\;\mathrm{in}\;\mathrm{microemulsion}}{\mathrm{Concentration}\;\mathrm{of}\;\mathrm{oil}\;\mathrm{loaded}\;\mathrm{in}\;\mathrm{the}\;\mathrm{microemulsion}}\times 100$$

### 2.6. Safety Study of P. frutescens, M. oleifera, and Mixed Seed Oil Microemulsions

Five male and five female healthy volunteers were recruited at a dermatology clinic at Thammasat University Hospital. All human research studies were approved by human research ethics committee 085/2020 of the Faculty of Medicine, Thammasat University. Inclusion criteria were male or female, aged between 20 to 60 years old, who were proved to be healthy from laboratory results. Body mass index (BMI) of healthy volunteers was in the range of 18.5–24.9 kg/m^2^. All volunteers voluntarily participated in the project and understood and were willing to follow the instructions during the study.

Exclusion criteria were having congenital disease or a history of heart disease, liver disease, kidney disease, immune-related diseases such as immunodeficiency, autoimmune disease, pregnant, or having a history of allergy to *P. frutescens*, *M. oleifera* seed oil, or other ingredients in the microemulsion. Subjects were excluded if they were using a medication, such as antihistamine, steroid, or immunosuppressant. Withdrawal or termination criteria were having adverse reaction or a serious drug-related condition, including swelling nausea, severe vomiting, chest tightness, wheezing, or liver enzyme (AST, ALT) and blood urea nitrogen (BUN) values being greater than 25 times of normal criteria or creatinine values being greater than 1.5 times of the normal criteria [20].

#### 2.6.1. PBMC Isolation

The blood from healthy volunteers was carefully layered over histopaque-1077 and centrifuged at 400× *g* for 30 min at room temperature. The upper layer was aspirated, leaving the mononuclear cell layer at the interphase. The mononuclear cell layer was transferred to a new conical tube and 10 mL of phosphate buffer was added. The supernatant was carefully removed followed by centrifugation at 250× *g* for 10 min. The supernatant was aspirated out. Cell pellets were resuspended with RPMI medium and centrifuged at 250× *g* for 10 min.

#### 2.6.2. Cytotoxicity Study of *P. frutescens*, *M. oleifera*, and Mixed Seed Oil Microemulsions against Peripheral Blood Mononuclear Cells

Peripheral blood mononuclear cells (PBMC) (100,000 cells/well) were cultured in RPMI medium supplemented with 10% FBS and 1% penicillin-streptomycin. Cells were plated in 96-well plates and incubated under 37 °C and 5% CO_2_ for 24 h. *P. frutescens*, *M. oleifera*, and mixed seed oil microemulsions were dissolved in medium at concentrations ranging from 3.91 to 500 µg/mL. After 24 h, 100 µL of microemulsions were added to the cells and incubated for another 24 h. Samples were removed and Prestoblue (Invitrogen, Waltham, MA, USA) was added and incubated at 37 °C and 5% CO_2_ for 30 min. Absorbance was measured at wavelengths of 570 nm and 600 nm by UV-visible spectrophotometer microplate reader (Vario skan flash, Thermo scientific, Waltham, MA, USA). The cell viability percentage was calculated by Equation (5), where the control was the viability of untreated cells. The IC50 was calculated based on the non-linear regression analysis.

(5) $$\mathrm{Cell}\;\mathrm{viability}\;(\%)=\frac{\left(A570-A600\;\mathrm{of}\;\mathrm{tested}\;\mathrm{cells}\right)}{\left(A570-A600\;\mathrm{of}\;\mathrm{control}\right)}\times 100$$

#### 2.6.3. Skin Irritation Test

Healthy volunteers were questioned, physical examined, and vital sign checked. Blood samples of 10 mL were drawn and evaluated for blood sugar level, complete blood cell count liver function values, renal function values, and blood lipid profiles. In addition, urine was collected and characterized for specific gravity and pH values. The volunteer’s upper back was checked to determine that there was no rash or blisters in any area, and it was wiped with 70% alcohol. The testing substances, including *P. frutescens*, *M. oleifera*, and mixed seed oil microemulsion, were applied to the upper back of volunteers under an occlusive patch for 48 h. After 48 h and 72 h, the patch sites were photographed, recorded, and graded according to the criteria of International Contact Dermatitis Research Group (ICDRG). The criteria followed +? = doubtful reaction, + = weak, positive reaction, ++ = strong positive reaction, +++ = extreme positive reaction, IR = irritant reaction, IE = negative reaction.

### 2.7. Formulation of Emulgels Containing P. frutescens, M. oleifera, and Mixed Seed Oil Microemulsion

Emulgels containing *P. frutescens*, *M. oleifera*, and mix seed oil microemulsion were prepared by dispersing PEG/PPG-14/7 dimethyl ether (2% *w*/*w*) in purified water. Xanthan gum (0.75% *w*/*w*) was separately dispersed in glycerin (10% *w*/*w*) and added into PEG/PPG-14/7 solution. The mixture was thoroughly mixed using a homogenizer (IKA^®^-Werke GmbH & Co. KG, Staufen, Germany) until the hydrogel was formed. The microemulsions of *P. frutescens*, *M. oleifera*, or mixed seed oil (35% *w*/*w*) and phenoxyethanol (1% *w*/*w*) were added to form emulgels.

### 2.8. Evaluation of the Physical Characteristics and Stability Testing of Emulgels Containing P. frutescens, M. oleifera, and Mixed Seed Oil Microemulsions

The pH of the emulgels was measured using a pH meter (Eutech Instruments, Singapore). The viscosity of the emulgels was measured using a viscometer (NDJ 85, Yanhe, China). The physical stability of emulgels containing microemulsions of *P. frutescens*, *M. oleifera*, and mixed seed oil was studied by the heating–cooling cycle stability study for 6 cycles. Emulgels containing three different types of microemulsions were stored in tight containers for 24 h at 4 °C and were placed in an incubator at 45 °C for another 24 h, accounting for 1 cycle, with 6 cycles in total [19]. The long-term stability of emulgels containing microemulsions of *M. oleifera* seed oil, *P. frutescens* seed oil, and mixed seed oil was investigated by storage of the emulgels at 4 °C, 30 °C, and 45 °C for 1 month. The pH and viscosity were measured at the end of each cycle and at 1-month storage.

### 2.9. Statistical Analysis

Statistical analysis of data was performed using an analysis of variance (one-way or two-way ANOVA), followed by Tukey’s as a post-hoc test to assess the significance of differences (GraphPad Prism, La Jolla, CA, USA). A value of *p* < 0.05 was considered statistically significant in all cases.

## 3. Results and Discussion

### 3.1. Plant Sterols of P. frutescens, M. oleifera, and Mixed Seed Oil

The concentration of total plant sterols in *P. frutescens* seed oil were 216.04 mg/100 g, containing 212.67 mg, β-sitosterol, and 3.37 mg campesterol (Table 1). These results agreed with previous reports that plant sterols mostly found in P. frutescents seed oil were β-sitosterol followed by campesterol [21,22,23]. *M. oleifera* seed oil contained 91.33 mg/100 g of total plant sterols, consisting of 54.48 mg β-sitosterol, 21.37 mg stigmasterol, 8.94 mg campesterol, and 0.54 mg brassicasterol. These results were confirmed by previous findings reporting that β-sitosterol, stigmasterol, and campesterol were the main components in *M. oleifera* seed oil [24,25,26]. However, the concentrations of three plant sterols found in this study were shown to be higher than other studies [24]. The difference of plant sterol concentrations depended on several factors, including plant growth environmental factor, plant harvesting time, types of fertilization, plant genetics, and plant growing condition [27]. Mixed seed oil contained 57.18 mg/100 g of total plant sterols and only β-sitosterol (57.18 mg) was detected. These results indicated that the mixed seed oil was comprised of a high amount of β-sitosterol, and the other plant sterols might be lower than the limit of detection or quantification of the analytical method. Although mixed seed oil contained only β-sitosterol, this plant sterol has several benefits for skin, especially for anti-inflammation. The phytosterol has been shown to retard leukocyte recruitment, reduce cytokines levels, and oxidative stress to inhibit inflammation in mouse model of acute inflammation [28]. Β-sitosterol obtained from plant seed oils exhibited anti-inflammatory activities by inhibiting the activation of ERK/p38 and NF-κB pathways in peritoneal macrophages [28,29]. Β-sitosterol displayed potent anti-inflammatory effects in rat model with paw edema by reducing the volume of pleural exudate and decreasing the number of neutrophils [30].

### 3.2. Fatty Acid Compositions of P. frutescens, M. oleifera, and Mixed Seed Oil

Fatty acid content analysis in *P. frutescens*, *M. oleifera*, and mixed seed oil showed that the unsaturated fatty acids in *P. frutescens* oil were alpha-linolenic acid (56.27 g/100 g), followed by linoleic acid (17.78 g/100 g) and cis-9-oleic acid (11.51 g/100 g) (Table 2). Other types of unsaturated fatty acids found in *P. frutescens* seed oil were lauric acid, myristic acid, and arachidic acid. The amounts of these unsaturated fatty acids in *P. frutescens* seed oil were comparable with other reports [21,31,32]. *M. oleifera* oil contained several types of fatty acids, including cis-9-oleic acid (66.03 g/100 g), palmitic acid (7.91 g/100 g), and behenic acid (5.91 g/100 g). The other fatty acids found in *M. oleifera* seed oil were stearic acid and arachidic acid. These results were consistent with the fatty acid profiles of *M. oleifera* seed oil in other sources, showing that oleic acid was the unsaturated fatty acid mostly found in *M. oleifera* seed oil [25,33,34]. In addition, *M. oleifera* seed oil also contained lignoceric acid (1.59%), which has never been reported in previous studies. Fatty acids in mixed seed oil were found to be cis-9-oleic acid (36.84 g/100 g), alpha-linolenic acid (31.71 g/100 g), and linoleic acid (9.96 g/100 g). Mixed seed oil consisted of saturated and unsaturated fatty acids from both *P. frutescens* and *M. oleifera* seed oil. However, some fatty acids existed in *P. frutescens* seed oil, including heptadecanoic acid, cis-11-eicosenoic acid, and erucic acid, which disappeared from the mixed seed oil. In the mixed seed oil, the amount of cis-9-oleic acid was comparable to alpha-linolenic acid, followed by linoleic acid.

### 3.3. Peroxide Value in P. frutescens, M. oleifera, and Mixed Seed Oil

*P. frutescens*, *M. oleifera*, and mixed seed oil had the peroxide values of 8.70, 5.90, and 10.86 mEq/kg, respectively, which met the oil standards of cold pressed extract, for which the peroxide value of the oil must not be more than 15 mEq/kg [35]. Peroxide value indicated the production of lipid oxidation [36]. Typically, peroxide values increased with an increment storage time of oils. The fatty acid composition also affected the oxidation rate and rancidity of the oil [37]. In general, increasing the amount of linolenic acid and/or decreasing the level of oleic acid caused a decrease in the oxidative stability of the oils [38]. Salama et al. demonstrated that pure *M. oleifera* seed oil had a low peroxide value as a result of the high content of oleic acid and low amount of linoleic and linolenic acid. This result agreed with our results, suggesting the lower peroxide value of *M. oleifera* seed oil compared with *P. frutescens* seed oil and mixed seed oil. Mixed seed oil contained a lower amount of oleic acid compared with *M. oleifera* seed oil and greater amounts of alpha-linolenic acid and linoleic acid resulting in the higher susceptibility to rancidity [39]. Compared to *P. frutescens* seed oil, the peroxide value of mixed seed oil was higher, probably due to the presence of other fatty acid, including palmitoleic acid, lauric acid, behenic acid, and lignoceric acid in the mixed seed oil.

### 3.4. Thiobarbituric Acid Test

Thiobarbituric acid is a reagent for the measurement of fat and oil oxidation. The TBA number represents the breakdown products of unsaturated fatty acid oxidation. The TBA number of *P. frutescens*, *M. oleifera*, and mixed seed oil were 3.94, 0.1, and 2.61 mg malonaldehyde/kg, respectively. The increase in the TBA number of a sample demonstrates the rancidity of the sample [40].

### 3.5. Color of P. frutescens, M. oleifera, and Mixed Seed Oil

The results of color measurement showed that L*, a*, and b* were positive values, suggesting that *M. oleifera* seed oil had a light yellow color, whereas *P. frutescens* seed oil was darker and more reddish. When the oils were mixed at a 1:1 ratio, the color was darker with more yellow and red colors (Table 3).

### 3.6. Formulation of P. frutescens, M. oleifera, and Mixed Seed Oil Microemulsions

In this study, the optimal proportion of surfactants and co-surfactants in microemulsion was determined by the construction of the pseudo-ternary phase diagrams using the phase titration method. The effects of different types of surfactants and the ratio of surfactant and co-surfactant on the microemulsion system were investigated. The ratio of surfactant, i.e., Tween 80 and Tween 20, and cosurfactant, i.e., propylene glycol, was kept constant at 1:1. Compared with other formulations, the optimal formulations were obtained by using Tween 80 as a surfactant only. Microemulsions were formed from the system composed of 0.91–45.45% *w*/*w P. frutescens* seed oil, 0.91–27.27% *M. oleifera* seed oil, and 1.43–81.82% of mixed seed oil, 9–81.81% *w*/*w* Tween 80, and 9.09–90.9% *w*/*w* water.

The criteria of selection of the optimal formulation were based on the transparency region, small size (<300 nm), narrow polydispersity index (<0.4 nm), and negative zeta potential value of microemulsion. The larger transparency region of microemulsion reflected the higher solubilizing capacity of microemulsion that enabled the increase in the solubility of oils or poorly water-soluble compounds and helped increase the partitioning of drugs into the stratum corneum [41]. The microemulsion prepared by using Tween 80 as a surfactant resulted in the largest transparent region (Supplementary Table S1). The microemulsion of mixed seed oil had the smallest size, the lowest PDI, and the negative charge of microemulsion, indicating the most optimal colloidal property (Supplementary Table S2).

The results showed that mixed oil microemulsion, using Tween 80 as a surfactant without co-surfactant, showed a larger transparent region compared with single oil. These results confirmed our previous report showing that Tween 80 alone produced the best formulation of microemulsion of *P. frutescens*, *M. oleifera*, and mixed seed oil [35]. The mechanism underlying stabilization of Tween 80 was due to the fact that Tween 80 acted as an emulsifier, adsorbing at the oil droplet surface [42].

Propylene glycol has been used as a co-surfactant to help dissolve oil in water. Incorporating propylene glycol into a surfactant layer may increase interfacial fluidity of microemulsion [43,44]. However, our results showed that mixing propylene glycol with Tween 20 or Tween 80 resulted in a lower transparent region and a separation of microemulsion into two phases within 1 week. The results suggested that adding propylene glycol to the system decreased the concentration of Tween 80 and hence destabilized the spontaneously formed microemulsion [45]. The surfactant directly plays an important role in the formation of microemulsion, whereas the co-surfactant improves the microemulsion stability by increasing fluidity and disordering degree on the surfactant film [46]. Date et al. suggested that due to its high polarity, the concentration of the co-surfactant should be minimized because it tended to migrate toward the aqueous phase upon dispersion into the aqueous medium and may lead to oil and water separation [47]. The microemulsions obtained from 25% Omix (12.5% of oil and 12.5% of Lexol^®^, 58.33% Tween 80, and 16.67% deionized water were selected for further characterization because this formulation contained the maximum amount of oil that was stable for at least 1 week of observation.

### 3.7. Appearance of P. frutescens, M. oleifera, and Mixed Seed Oil Microemulsion

**Figure 1:**
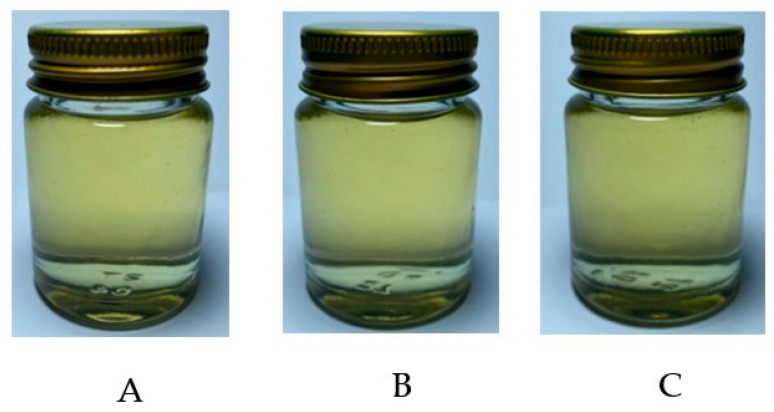
Appearance of (**A**) *P. frutescens* (**B**) *M. oleifera*, and (**C**) mixed seed oil microemulsions after fresh preparation.

The appearance of *P. frutescens*, *M. oleifera*, and mixed seed oil microemulsions after fresh preparation is shown in Figure 1. Three types of microemulsion had a clear yellow color and were homogeneous.

### 3.8. Size, Size Distribution, and Surface Charge of P. frutescens, M. oleifera, and Mixed Seed Oil Microemulsion

**Figure 2:**
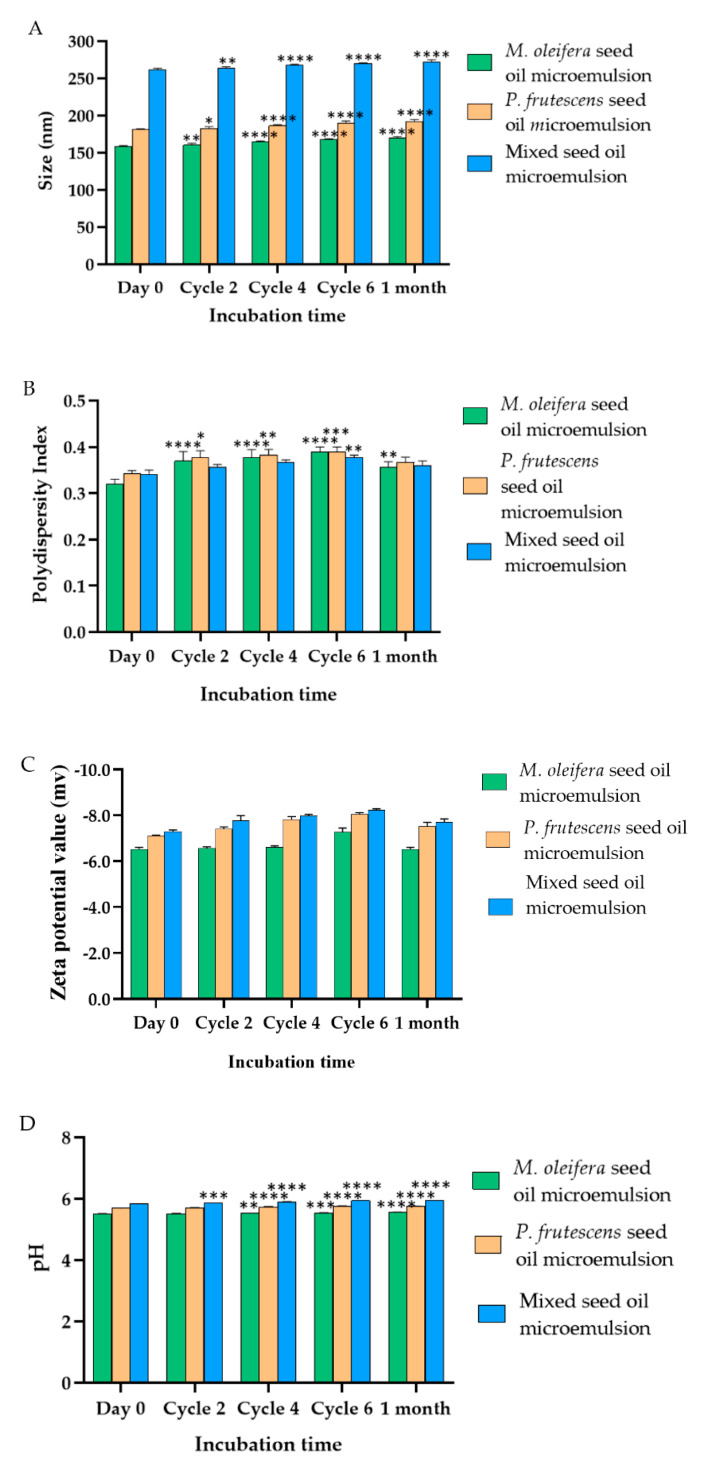
(**A**) Particle size, (**B**) polydispersity index, and (**C**) zeta potential values. (**D**) pH of freshly prepared microemulsion of *P. frutescens*, *M. oleifera*, and mixed seed oil microemulsion and microemulsions after 2, 4, and 6 cycles of heating–cooling stability test and 1 month storage at 30 °C. Data represent mean ± SD (n = 3), * indicates *p* < 0.05, ** indicates *p* < 0.01, *** indicates *p* < 0.001, and **** indicates *p* < 0.0001, compared with Day 0.

The average droplet size, polydispersity index, zeta potential, and pH of *P. frutescens*, *M. oleifera*, and mixed seed oil microemulsions after fresh preparation, heating–cooling cycle stability test, and long-term storage at 30 °C for 1 month are expressed in Figure 2. *P. frutescens*, *M. oleifera*, and mixed seed oil microemulsions had an average size of 181 ± 1 nm, 158.33 ± 1.53 nm and 261.67 ± 2.08 nm, respectively. These results were confirmed by our previous report [35]. The polydispersity index of *P. frutescens*, *M. oleifera*, and mixed seed oil microemulsions were 0.34 ± 0.005, 0.32 ± 0.01, and 0.34 ± 0.01, respectively. The polydispersity index is used to describe the degree of non-uniformity of a size distribution of microemulsion. Polydispersity can occur due to size distribution in a sample or agglomeration or aggregation of the sample during isolation or analysis [48]. *P. frutescens*, *M. oleifera*, and mixed seed oil microemulsions had zeta potential values between −6.5 and −8.2 mV. The zeta potential values of microemulsions were slightly negative due to the fatty acid components in the oils. This negative charge helped stabilize the microemulsion system by producing electrostatic repulsive forces of head groups which thereby hinder aggregation with nearby droplets [49]. The zeta potential values of all microemulsions were not significantly changed upon the stability study, indicating the stability of Tween 80 adsorption on the surface of oil droplets. The pH values of all microemulsions ranged between 5.50 ± 0.02 and 5.83 ± 0.01, which was in the normal pH range of skin, suggesting that microemulsions may not irritate human skin [50]. However, some surfactants can cause irritation when applied to the skin when Tween 80 concentration in the mixture was up to 58.33%. Therefore, it was necessary to evaluate the safety of all microemulsions. The stability studies revealed that the size, polydispersity index, and pH value of microemulsion slowly increased from the baseline. However, these values were acceptable. The stability assessment suggested that all microemulsions can be stored at 30 °C for up to 1 month.

### 3.9. Physical Stability of P. frutescens, M. oleifera, and Mixed Seed Oil Microemulsions

**Figure 3:**
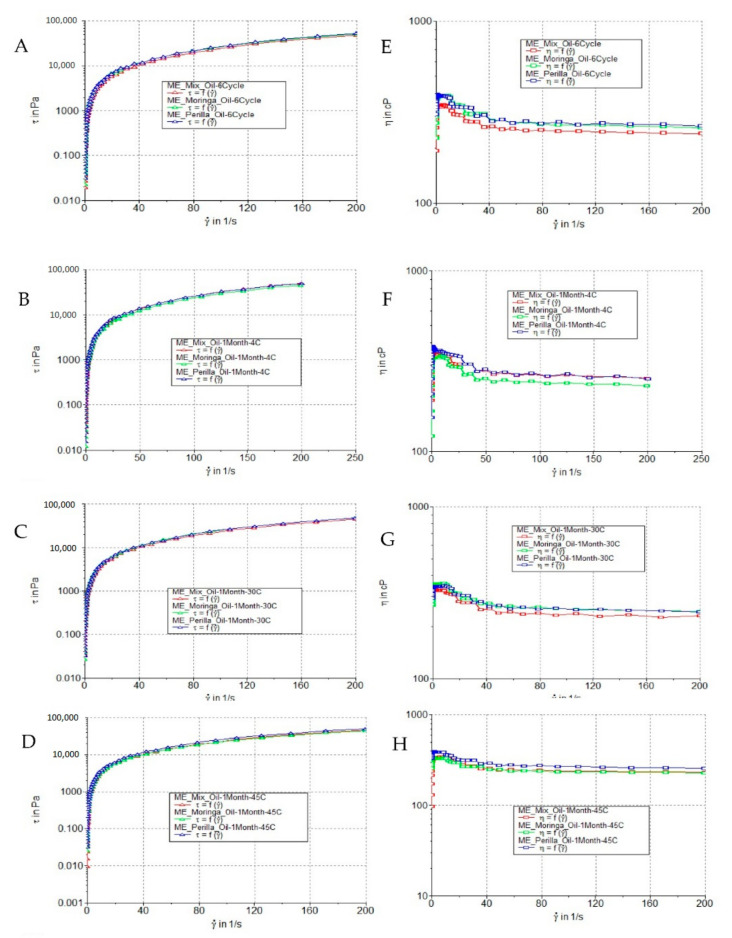
Flow curves of *P. frutescens*, *M. oleifera*, and mixed seed oil microemulsions measured at day 0 after storage at (**A**) six cycles of heating–cooling stability test. (**B**) 4 °C, (**C**) 30 °C, and (**D**) 45 °C, expressed as shear rate and applied shear stress. Flow curves of *P. frutescens*, *M. oleifera*, and mixed seed oil microemulsions at day 0 after storage at (**E**) six cycles of heating–cooling stability test (**F**) 4 °C, (**G**) 30 °C, and (**H**) 45 °C, expressed as viscosity and shear rate.

The rheological data exhibited non-linear proportionality between shear rate and shear stress, suggesting that all microemulsions displayed non-Newtonian fluid behavior (Figure 3). It was observed that when increasing shear rate, the viscosity of microemulsions decreased, indicating that the microemulsions had shear-thinning or pseudoplastic flow behavior [51]. *M. oleifera* seed oil showed the highest viscosity, followed by *P. frutescens* seed oil and mixed seed oil. This result agreed with the viscosity of the oils. The viscosity values of *P. frutescens* seed oil, *M. oleifera* seed oil, and mixed seed oil were 29.56 ± 0.29, 44.86 ± 0.00, and 43.40 ± 0.29 kcPs, respectively. After heating–cooling tests at all six cycles and 1 month storage at 4 °C, 30 °C, and 45 °C, the appearance, color, odor, and texture of the microemulsion were not changed. When compared with freshly prepared microemulsions, the viscosity of all microemulsions was not significantly changed, except *P. frutescens* seed oil microemulsion stored at 45 °C, as shown in Table 4.

### 3.10. Loading Efficiency and Chemical Stability of P. frutescens, M. oleifera, and Mix Seed Oil Microemulsions

**Figure 4:**
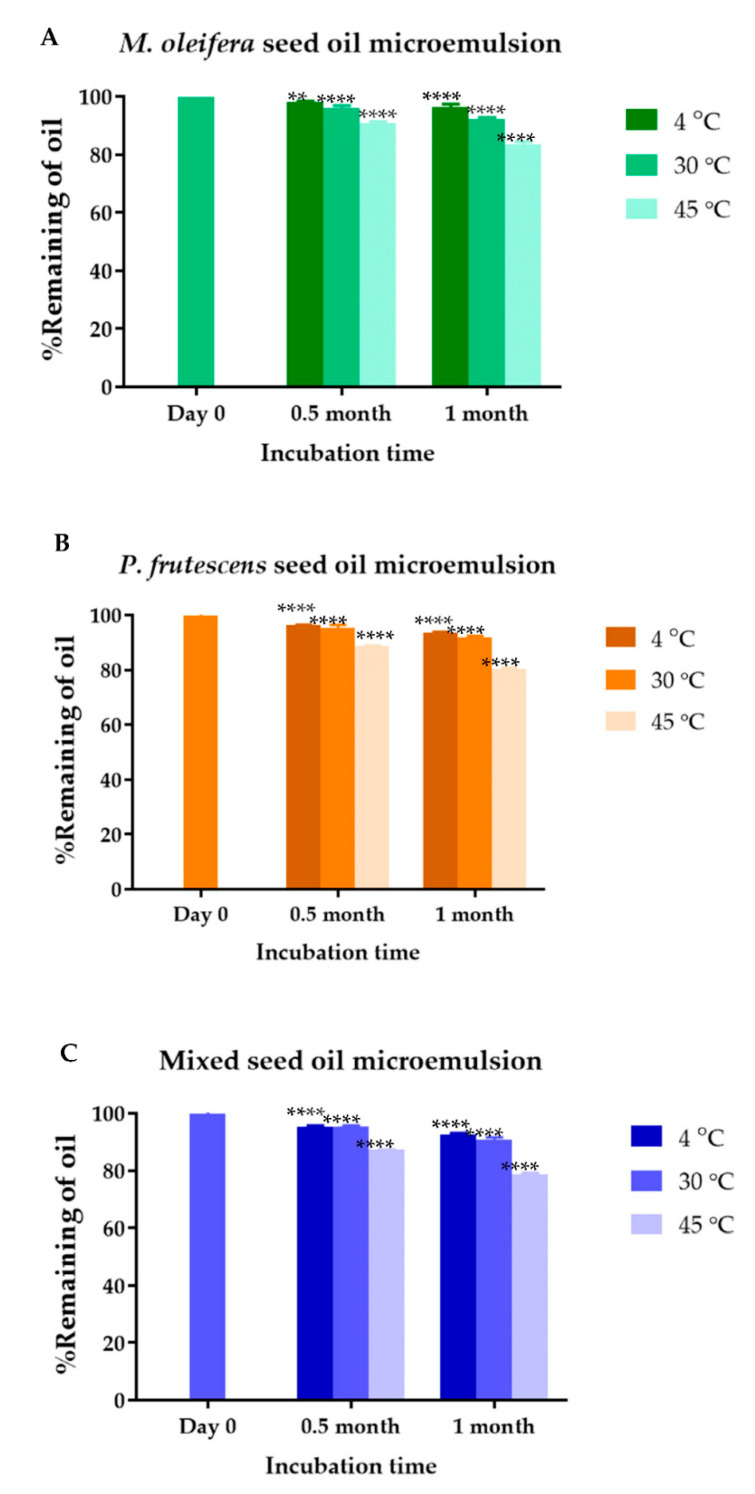
Chemical stability of oil in *P. frutescens*, *M. oleifera*, and mix seed oil microemulsions. Data represent mean ± SD from three experiments. ** indicates *p* < 0.01, and **** indicates *p* < 0.0001, compared with Day 0.

The UV-vis spectrophotometry of *P. frutescens*, *M. oleifera*, and mixed seed oil microemulsions showed the pronounced peaks at 285 nm, 259 nm, and 275.5 nm, respectively. The absorbance values increased with the concentration of the oils. *P. frutescens* seed oil, *M. oleifera* seed oil, and mixed seed oil standard equations were y = 0.0841x + 0.0089, y = 0.1312x + 0.0521, and y = 0.1229x + 0.0137, respectively. The concentrations of *P. frutescens*, *M. oleifera*, and mixed seed oils loaded into microemulsions were 12.5% *v*/*v*. The calculated loading efficiency of *P. frutescens*, *M. oleifera*, and mixed seed oil microemulsion were 99.93 ± 0.13%, 99.98 ± 0.02%, 99.88 ± 0.19%, respectively. The chemical stability of *P. frutescens*, *M. oleifera*, and mixed seed oil microemulsion was shown in Figure 4. The % remaining of *P. frutescens* seed oil in microemulsion stored at 4 °C, 30 °C, and 45 °C for 1 month significantly decreased to 93.59 ± 0.39, 91.93 ± 0.48, and 80.46 ± 0.43, respectively. *M. oleifera* seed oil in microemulsion stored at 4 °C, 30 °C, and 45 °C for 1 month remained 96.45 ± 0.95, 92.22 ± 0.54, and 83.43 ± 1.06%, respectively. Mixed seed oil in microemulsion stored at 4 °C, 30 °C, and 45 °C for 1 month contained 92.68 ± 0.39, 90.85 ± 0.80, and 78.70 ± 0.54% of oil, respectively. *M. oleifera* seed oil in the microemulsion had greater chemical stability compared to other microemulsions. Ogunsina et al. showed that cold pressed *M. oleifera* seed oil had good thermal and oxidative stability, which was a result of high oleic acid content [52]. The mechanism of oxidation in oils was based on a lipid peroxidation reaction. Because *P. frutescens* seed oil contained high concentrations of alpha-linolenic acid and linoleic acid, it was vulnerable to oxidative degradation. Therefore, mixing *P. frutescens* seed oil with *M. oleifera* seed oil might strengthen the resistance to oxidation and improve pharmacological properties compared with the starting single oils [17]. The solubility and stability of oils might be improved by the preparation of a microemulsion based drug delivery system by forming micelles with the surfactant. The increase in stability of oil in a microemulsion-based formulation has been reported [53,54,55]. The volatile components in *P. frutescens* and *M. oleifera* seed oil microemulsions, containing alkanes, carboxylic acids, and esters, might vaporize more readily at 45 °C compared to at lower temperatures. Therefore, it was suggested to store all microemulsions at 4 °C or 30 °C.

### 3.11. Cytotoxicity of P. frutescens, M. oleifera, and Mixed Seed Oil Microemulsions against Peripheral Blood Mononuclear Cells

**Figure 5:**
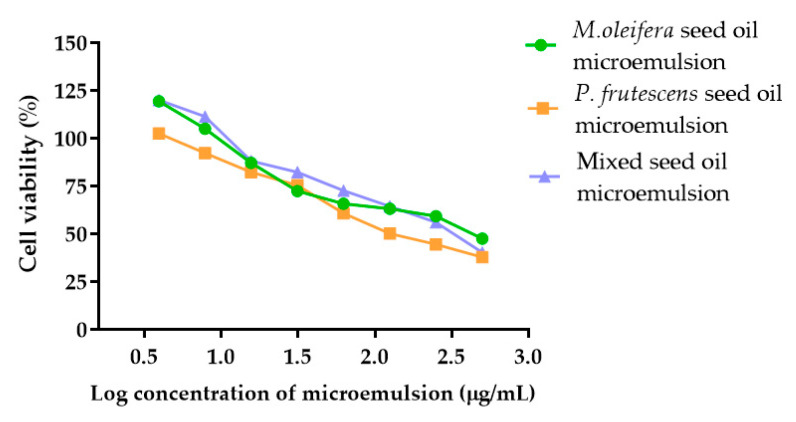
Cell viability percentage of PBMC in the presence of *P. frutescens, M. oleifera*, and mixed seed oil microemulsions at various concentrations. Data represent mean ± SD from three experiments.

Because emulgels containing *P. frutescens* seed oil microemulsion, *M. oleifera* seed oil microemulsion, and mixed seed oil microemulsion may penetrate skin and were absorbed to peripheral blood, PBMCs might be prone to exposure to the microemulsions. Therefore, the toxicity of microemulsions against PBMCs was investigated. Several applications of PBMCs in toxicology have been reported, including new compound toxicity assessment, especially on the human immune system and the dosage limit of new drug determination [56]. In this study, the effects of *P. frutescens*, *M. oleifera*, and mixed seed oil microemulsions on PBMC cytotoxicity were investigated to select the optimal concentration of the microemulsions for product development. Cells were treated with three types of microemulsions in the concentration range of 3.91–500 µg/mL. The increase in the concentration of *P. frutescens*, *M. oleifera*, and mixed seed oil microemulsions decreased the cell viability in a dose-dependent manner (Figure 5). The IC50 values of *P. frutescens*, *M. oleifera*, and mixed seed oil microemulsions against PMBC were 166.7, 331.8, and 284.3 µg/mL, respectively. The results indicated that *P. frutescens* seed oil was more toxic than *M. oleifera* seed oil and mixed seed oil, respectively. Fatty acids have been shown to induce inflammatory gene expression in PBMCs, with or without lipopolysaccharide stimulation [57]. Saturated fatty acids, including palmitic acid, and unsaturated fatty acid, such as gamma-linoleic acid and arachidonic acid, elicited inflammatory gene expression in PBMC, whereas oleic acid, alpha-linolenic acid, and docosahexaenoic acid reduced inflammatory gene expression [58].

### 3.12. Safety Evaluation of Microemulsions in Healthy Volunteers

Fifteen volunteers were included in the safety evaluation study. Five volunteers were excluded due to the high LDL values. Therefore, there were 10 volunteers divided into 5 females and 5 males. From the data analysis, the age of the subjects ranged from 25 to 44 years old, and the average of age was 31.3 ± 7.11 years. Because *P. frutescens*, *M. oleifera*, and mixed seed oil microemulsions were mainly composed of saturated and unsaturated fatty acids, laboratory blood tests were performed to determine fasting blood sugar, complete blood count, liver function, renal function, and lipid profiles in volunteers. Comparing the results of laboratory blood tests before and after the study, it was found that there was no change in the laboratory testing of all 10 volunteers (Table 5). The safety of *P. frutescens* seed oil has been reported in rodents and beagle dogs. No significant treatment-associated toxicity or mortality was observed, suggesting that *P. frutescens* seed oil was well tolerated in animal models. The tolerated doses of beagle dogs, mice, and rats were 3 g/kg/day, 50 g/kg, and 20 g/kg, respectively [59]. Oral administration of 500 mg two times/day for six months showed no significant change in complete blood counts, kidney function tests, or liver function panels between baseline and after treatment [60]. All types of microemulsions did not induce skin irritation and allergic reactions after 48 and 72 h of application. These results imply that *P. frutescens*, *M. oleifera*, and mixed seed oil microemulsions were safe for applying to skin. It may be concluded that *P. frutescens*, *M. oleifera*, and mixed seed oil microemulsions were highly safe and were suitable for use as topical products for the skin. Athikomkulchai et al. reported that cream containing *M. oleifera* seed oil did not induce irritation on the skin of thirty two healthy volunteers [61].

The topical administration of active compounds is impaired by limited skin permeability due to the presence of skin barriers. Microemulsions are a lipid-based drug delivery system with high potential to increase drug permeation through the skin. This study revealed the feasibility of formulating three types of microemulsions containing *P. frutescens* seed oil, *M. oleifera* seed oil, and mixed seed oil. The microemulsions developed in this study offer industrial importance in terms of spontaneous formation, ease of manufacturing and scale-up, thermodynamical stability, and cost-effectiveness [62]. In the clinical aspect, the microemulsions of three types of oils revealed the possibility of the therapeutic use because they do not induce skin irritation on human volunteers and the microemulsions were not toxic to PBMCs, indicating low cytotoxicity and immunogenicity [63].

### 3.13. Physical Characteristics and Stability of Emulgels Containing P. frutescens, M. oleifera, and Mixed Seed Oil Microemulsion

**Figure 6:**
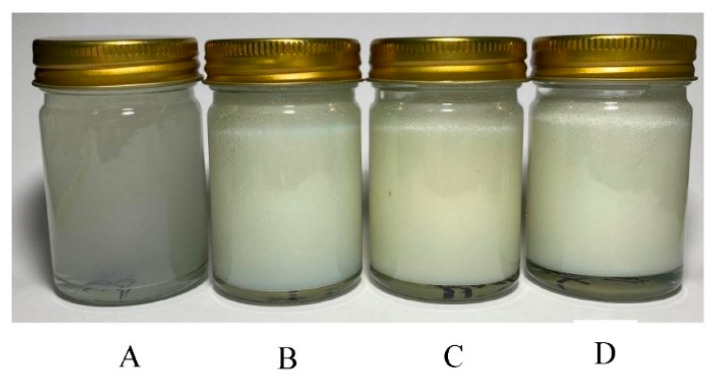
Appearance of (**A**) emulgel base, (**B**) emulgel containing *P. frutescens* seed oil microemulsion, (**C**) emulgel containing *M. oleifera* seed oil microemulsion, and (**D**) emulgel containing mixed seed oil microemulsion.

**Figure 7:**
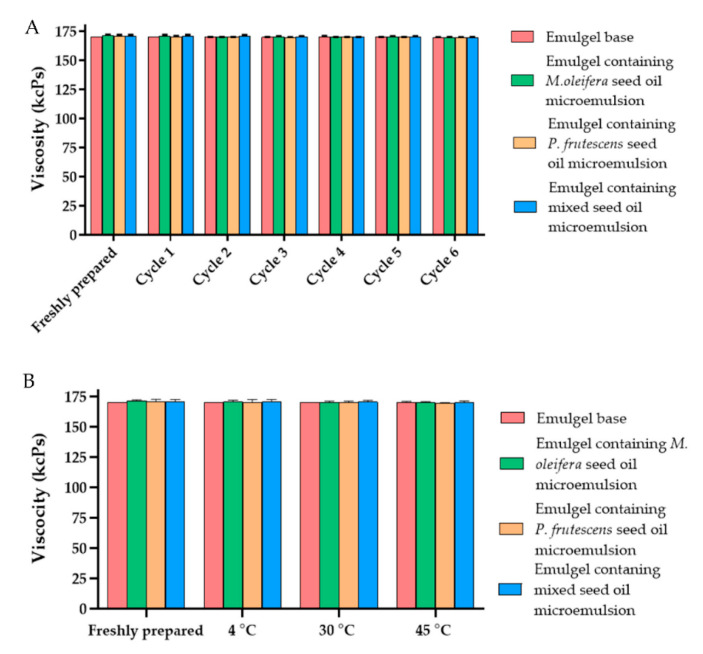
(**A**) Effects of six heating—cooling cycle stability tests on the viscosity of emulgel base and emulgels containing *P. frutescens*, *M. oleifera*, and mixed seed oil microemulsion. (**B**) Effect of temperature on the viscosity of gel base and emulgels containing *P. frutescens*, *M. oleifera*, and mixed seed oil microemulsion after storage at 4 °C, 30 °C, and 45 °C for 1 month. Data represent mean ± SD from three experiments.

**Figure 8:**
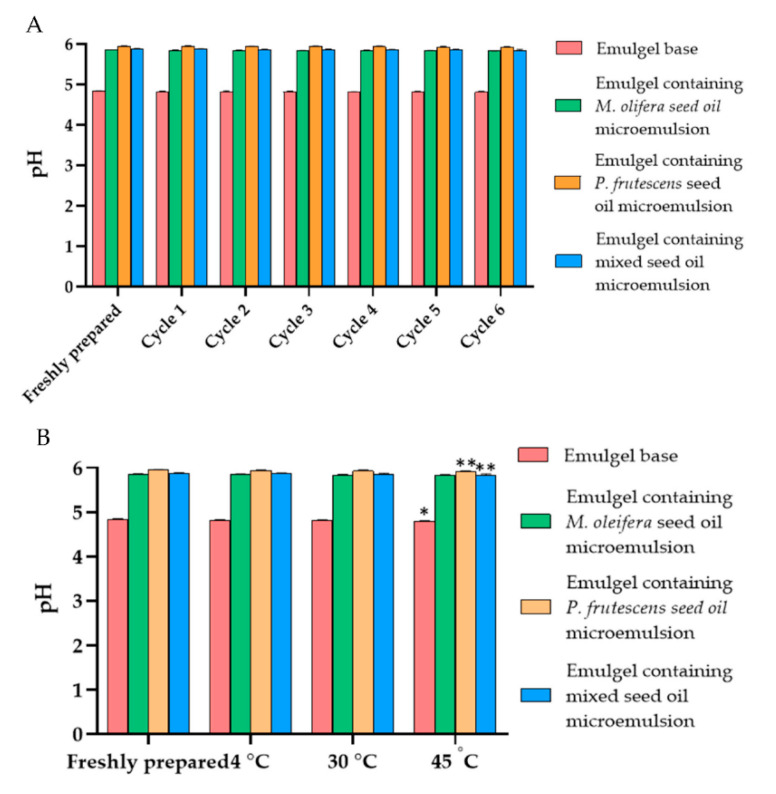
(**A**) pH values of emulgel base, emulgel containing *P. frutescens*, *M. oleifera*, and mixed seed oil microemulsion after six heating–cooling cycles stability tests. (**B**) pH values of emulgel base and emulgels containing *P. frutescens*, *M. oleifera*, and mixed seed oil microemulsion after storage at 4 °C, 30 °C, and 45 °C for 1 month. Data represent mean ± SD from three experiments. * indicates *p* < 0.05, and ** indicates *p* < 0.01, compared with freshly prepared emulgel.

The physical characteristics of emulgels containing *P. frutescens*, *M. oleifera*, and mixed seed oil microemulsion were shown in Figure 6. The microemulsion incorporated emulgels were yellowish with a smooth texture and the oil had a distinctive odor. The color and odor of the emulgels were not changed from the initial preparation and they were honogenous without separation after stability studies. The viscosity of emulgel base, emulgels containing *P. frutescens*, *M. oleifera*, and mixed seed oil microemulsions after preparation were 170.2 ± 0.0, 170.7 ± 1.21, 171.66 ± 0.58, and 171.1 ± 1 kcPs, respectively. The viscosity of emulgels after six heating–cooling cycle stability tests and after storage at 4, 30, and 45 °C for 1 month did not alter from the baseline (Figure 7). The emulgels containing *P. frutescens*, *M. oleifera*, and mixed seed oil microemulsion had pH values of 5.95 ± 0.01, 5.86± 0.006, and 5.88 ± 0.01, respectively. The pH values of all emulgels were not changed after the stability test, suggesting that time and temperature did not affect the pH of the emulgels (Figure 8). The gel base had the pH value of 4.83 ± 0.01. The higher pH values of emulgels containing microemulsion compared with the gel base were influenced by the pH of *P. frutescens*, *M. oleifera*, and mixed seed oils, i.e., 5.77, 5.69, 5.97, respectively.

## 4. Conclusions

In the present study, *P. frutescens* seed oil, *M. oleifera* seed oil, and mixed seed oil microemulsions were successfully formulated to achieve the desired solubility and stability of the oil. Microemulsions exhibited acceptable physical and chemical stability. The safety of microemulsions was supported by the cytotoxicity of microemulsions to PBMC, laboratory test, and the non-irritation of human skin. Emulgels containing microemulsions were developed and showed good physical characteristics and stability. All types of emulgels were recommended to be stored at 4 °C or 30 °C. Therefore, three types of emulgels could be a potential alternative for delivery of *P. frutescens* seed oil, *M. oleifera* seed oil, and mixed seed oil.

## Figures and Tables

**Table 1:** Plant sterols in *P. frutescens* seed oil, *M. oleifera* seed oil, and mixed seed oil.

Compound	Content (mg/100 g)		
	*P. frutescens* Seed Oil	*M. oleifera* Seed Oil	Mixed Seed Oil
β-sitosterol	212.67	54.48	57.18
Campesterol	3.37	8.94	-
Stigmasterol	-	21.37	-
Brassicasterol	-	0.54	-

**Table 2:** Fatty acid composition of *P. frutescens* seed oil, *M. oleifera* seed oil, and mixed seed oil.

	*P. frutescens* Seed Oil	*M. oleifera* Seed Oil	Mixed Seed Oil
Saturated fatty acid (g/100 g)			
Lauric acid (C12:0)	0.16	-	0.06
Myristic acid (C14:0)	0.15	0.19	0.12
Palmitic acid (C16:0)	6.95	7.91	6.78
Heptadecanoic acid (C17:0)	-	0.10	-
Stearic acid (C18:0)	2.63	4.88	4.19
Arachidic acid (C20:0)	0.15	3.19	1.85
Behenic acid (C22:0)	-	5.91	2.84
Lignoceric acid (C24:0)	-	1.59	0.62
Unsaturated fatty acid (g/100 g)			
Palmitoleic acid (C16:1)		1.27	0.62
Cis-9-Oleic acid (C18:1n9c)	11.51	66.03	36.84
Linoleic acid (C18:2n6c)	17.78	1.53	9.96
Alpha-Linolenic acid (C18:3n3)	56.27	0.25	31.71
Cis-11-Eicosenoic acid (C20:1n11)	-	2.38	-
Erucic acid (C22:1n9)	-	0.15	-

**Table 3:** Results of color analysis of *P. frutescens, M. oleifera*, and mixed seed oils.

Color	*P. frutescen* Seed Oil	*M. oleifera* Seed Oil	Mixed Seed Oil
L*	49.96 ± 0.04	61.94 ± 27.11	48.43 ± 0.13
a*	2.51 ± 0.02	1.42 ± 5.77	3.25 ± 0.06
b*	61.47 ± 0.18	64.17 ± 2.04	65.55 ± 0.31

**Table 4:** Viscosity values of *P. frutescens*, *M. oleifera*, and mixed seed oil microemulsions freshly prepared, tested for six heating–cooling cycles, and 1 month storage at 4 °C, 30 °C, and 45 °C. ** indicates *p* < 0.01.

Formula	Viscosity (cPs)				
	Day 0	6 Cycles	4 °C 1 Month	30 °C 1 Month	45 °C 1 Month
*M.oleifera* oil microemulsion	257.43 ± 12.07	259.25 ± 1.33	231.95 ± 1.96	250.21 ± 6.68	213.41 ± 21.81
*P. frutescens* oil microemulsion	233.23 ± 38.89	251.46 ± 18.59	252.58 ± 1.66	240.81 ± 3.18	252.99 ± 5.42 **
Mix seed oil microemulsion	227.75 ± 15.07	238.17 ± 3.47	244.74 ± 10.69	225.69 ± 7.47	233.55 ± 2.70

**Table 5:** The laboratory results of healthy volunteers prior to and after treatment with three types of microemulsions.

Parameter	Before Test	After Test	Units	*p*-Value
Glucose	86.6 ± 7.17	88.9 ± 3.14	mg/dL	0.3037
Fasting blood sugar				
**Complete Blood Count**				
Red blood cell (RBC)	4.86 ± 0.76	4.94 ± 0.95	10^6^/cumm.	0.4288
White blood cell (WBC)	5.79 ± 1.33	5.38 ± 1.55	10^6^/cumm.	0.4689
Neutrophils	54.38 ± 8.71	58.34 ± 10.27	%	0.2608
Lymphocytes	37.25 ± 10.56	34.83 ± 8.15	%	0.436
Monocytes	3.41 ± 1.81	3.91 ± 2.00	%	0.5701
Basophils	0.54 ± 0.22	0.54 ± 0.46	%	0.1864
Eosinophils	3.24 ± 1.48	2.37 ± 1.48	%	0.1223
Hemoglobin (Hb)	13.39 ± 1.52	13.35 ± 1.79	mg/dL	0.899
Hematocrit (HCT)	41.77 ± 4.96	40.18 ± 4.09	%	0.0876
Platelet	268.4 ± 46.60	275.5 ± 49.15	K/cumm.	0.3017
**Liver Function**				
Total protein	7.91 ± 0.39	7.69 ± 0.40	g/dL	0.0955
Albumin	4.31 ± 0.29	4.33 ± 0.26	g/dL	0.764
Globulin	3.47 ± 0.28	3.39 ± 0.28	g/dL	0.0528
Alkaline phosphate	59.8 ± 13.43	59.1 ± 15.50	U/L	0.7886
Total bilirubin	0.47 ± 0.27	0.53 ± 0.35	mg/dL	0.4826
Conjugate bilirubin	0.14 ± 0.05	0.15 ± 0.07	mg/dL	0.5911
Serum glutamic oxaloacetic transaminase (SGOT)	17.1 ± 7.51	15.2 ± 3.49	U/L	0.3036
**Serum Glutamic** Pyruvate Transaminase (SGPT)	26.1 ± 4.31	26.6 ± 3.89	U/L	0.0522
**Renal Function**				
Blood urea nitrogen (BUN)	12.34 ± 1.95	11.55 ± 3.38	mg/dL	0.348
Creatinine	0.838 ± 0.23	0.863 ± 0.22	mg/dL	0.1636
HDL	62.9 ± 17.64	58.5 ± 9.65	mg/dL	0.3881
Cholesterol	209.2 ± 42.49	189.5 ± 29.55	mg/dL	0.2328
LDL	119.3 ± 27.37	114.3 ± 30.24	mg/dL	0.5782
Triglycerides	57.9 ± 24.70	59.3 ± 15.90	mg/dL	0.8806
**Urine Analysis**				
pH	6.0 ± 0.75	6.35 ± 0.71		0.0886
Specific gravity	1.03 ± 0.003	1.027 ± 0.003		0.0811

## Data Availability

Not applicable.

## Supplementary Materials

The following supporting information can be downloaded at: https://www.mdpi.com/article/10.3390/polym14122348/s1, Table S1: Percentages of oil and surfactant contained in the transparency region of pseudoternary phase diagrams; Table S2: Size, polydispersity index, and zeta potential values of mixed seed oil microemulsion.
