# Supplementary material for: Emulgels Containing Perilla frutescens Seed Oil, Moringa oleifera Seed Oil, and Mixed Seed Oil: Microemulsion and Safety Assessment
Source: Polymers (Basel). 2022 Jun 9;14(12):2348. doi: 10.3390/polym14122348 (PMC9231324; doi:10.3390/polym14122348)
Supplement: Supplementary file 1 [file polymers-14-02348-s001.zip › polymers-1758132-supplementary.pdf]

Table S1. Percentages of oil and surfactant contained in the transparency region of pseudoternary phase diagrams

Surfactant/co-surfactant	*P. frutescens* seed oil	*M. oleifera* seed oil	Mixed seed oil (%)
Tween 80	0.91-45.45	0.91-27.27	0.90-81.81
Tween 80 + PG	9.09-45.45	9.09-18.18	9.09-81.81
Tween 80 + Span 80	9.09-45.45	9.09-81.82	7.14-81.81
Tween 20	7.14-81.82	7.79-81.82	6.25-58.33
Tween 20 + PG	7.69-81.82	9.09-18.18	7.14-81.81
Tween 20 + Span 80	7.69-81.82	7.69-81.82	7.69-81.81

Table S2. Size, polydispersity index, and zeta potential values of mixed seed oil microemulsion

Formulation	Mixed seed oil (%)	Surfactants/co-surfactants				Deionized water (%)	Size (nm)	PDI	Zeta potential (mV)
		Tween 80 (%)	PG (%)	Span 80 (%)	Tween 20 (%)				
1	25	58.33	-	-	-	16.67	180.33±3.72	0.31±0.33	-7.49±0.57
2	25	29.17	29.17	-	-	16.67	230.28±4.21	0.50±0.04	-9.08±1.29
3	25	29.17	-	29.17	-	16.67	450.73±2.81	0.74±0.21	-7.32±1.36
4	25	-	-	-	58.33	16.67	400.32±0.89	0.55±0.03	-7.49±0.51
5	25		29.17		29.17	16.67	337.16±2.66	0.48±0.07	-7.89±0.74
6	25			29.17	29.17	16.67	585.20±-3.87	0.75±0.05	-7.88±2.23
